# Characterization of Diverse Anelloviruses, Cressdnaviruses, and Bacteriophages in the Human Oral DNA Virome from North Carolina (USA)

**DOI:** 10.3390/v15091821

**Published:** 2023-08-26

**Authors:** Elise N. Paietta, Simona Kraberger, Joy M. Custer, Karla L. Vargas, Claudia Espy, Erin Ehmke, Anne D. Yoder, Arvind Varsani

**Affiliations:** 1 Department of Biology, Duke University, Durham, NC 27708, USA; 2 The Biodesign Center for Fundamental and Applied Microbiomics, Center for Evolution and Medicine and School of Life Sciences, Arizona State University, Tempe, AZ 85287, USA; 3 Duke Lemur Center, Duke University, Durham, NC 27705, USA; erin.ehmke@duke.edu; 4 Structural Biology Research Unit, Department of Integrative Biomedical Sciences, University of Cape Town, Cape Town 7925, South Africa

**Keywords:** passive drool, *Anelloviridae*, *Redondoviridae*, *Cressdnaviricota*, *Caudoviricetes*, *Microviridae*, *Inoviridae*

## Abstract

The diversity of viruses identified from the various niches of the human oral cavity—from saliva to dental plaques to the surface of the tongue—has accelerated in the age of metagenomics. This rapid expansion demonstrates that our understanding of oral viral diversity is incomplete, with only a few studies utilizing passive drool collection in conjunction with metagenomic sequencing methods. For this pilot study, we obtained 14 samples from healthy staff members working at the Duke Lemur Center (Durham, NC, USA) to determine the viral diversity that can be identified in passive drool samples from humans. The complete genomes of 3 anelloviruses, 9 cressdnaviruses, 4 *Caudoviricetes* large bacteriophages, 29 microviruses, and 19 inoviruses were identified in this study using high-throughput sequencing and viral metagenomic workflows. The results presented here expand our understanding of the vertebrate-infecting and microbe-infecting viral diversity of the human oral virome in North Carolina (USA).

## 1. Introduction

The human oral cavity is teeming with diverse, abundant communities of prokaryotes, eukaryotes, and viruses [1]. These microbial communities are highly specialized to within-mouth niches, such as saliva and dental plaques [2,3]. Moreover, these communities have been found to contain microbe-infecting viruses (e.g., bacteriophages, redondoviruses), vertebrate-infecting viruses implicated in disease (e.g., Epstein–Barr virus, enteroviruses), and vertebrate-infecting viruses not currently implicated in disease (e.g., anelloviruses) [3,4,5,6,7,8]. Although not directly associated with disease, microbe-infecting viruses are relevant to human health as they alter microbial abundance, diversity, and evolution within the body [5,9]. Thus, understanding the diversity and evolutionary history of both vertebrate-infecting and microbe-infecting viruses of the human oral virome will have connections to human health and disease in the broadest sense.

While projects such as the Cenote Human Virome Database (CHVD) and Oral Virus Database (OVD) have been successful at pooling thousands of oral viral sequences and genomes based on host geography, our body of knowledge about the human oral virome is notably incomplete though rapidly expanding [1,3,10]. This rapid growth is largely due to the use of varying sample collection methods in conjunction with increasingly popular metagenomics surveys. Passive drool collection techniques are commonly used for extracting high-quality genomic data from saliva [11]. This approach collects unstimulated saliva, thus reducing contamination associated with spitting, swabbing, and rinsing [11,12,13]. Although some studies have looked at the oral microbiome and passive drool techniques for bacterial composition, this has not been the case for the oral virome where most studies have either used swabbing techniques or have not described a specific collection technique [4,14,15]. The few studies that have used unstimulated saliva collection methods combined with viral metagenomics have shown success in detecting viruses across numerous viral families [5]. Even so, there is a notable geographic bias in these studies. For saliva samples taken in the United States of America (USA), the OVD includes virus sequences or genomes from just 109 individuals with 101 of these individuals sampled in northern California [3,16,17]. Consequently, there is a scarcity of data on the human oral virome for most of the USA. To assess the feasibility of identifying complete virus genomes in saliva samples of humans, we tested passive drool collection and used viral metagenomic sequencing methods to characterize viruses in human saliva from the southeastern USA, focusing on the staff at a captive-primate colony as a pilot project. This study is part of a larger project identifying viruses in humans and nonhuman primates at the Duke Lemur Center (Durham, NC, USA).

## 2. Materials and Methods

### 2.1. Sample Collection

Saliva samples were collected from healthy adult participants using the passive drool method and Saliva Collection Aid (Salimetrics, Carlsbad, California, USA). These passive drool techniques where the participant allows saliva to pool in the mouth and drip into the collection aid allow for easy self-collection of up to ~2 mL of whole saliva while minimizing contamination [11,13]. Fourteen saliva samples were obtained from individual participants working or volunteering at the Duke Lemur Center between August 2021 and May 2022 (*n* = 14) (Durham, NC, USA). Samples were frozen at −80 °C until viral DNA extraction. This study was approved by the Duke University Campus Institutional Review Board (IRB #2022-0009).

### 2.2. Viral Nucleic Acid Extraction, Sequencing, De Novo Assembly, and Virus Genome Identification

Viral DNA was extracted from 200 µL of passive drool sample from the participants individually using the High Pure Viral Nucleic Acid Kit (Roche Diagnostics, Indianapolis, IN, USA). Rolling circle amplification was performed using the Illustra TempliPhi Kit (GE Healthcare, Chicago, IL, USA) to preferentially amplify circular DNA in the samples. Illumina sequencing libraries were generated using the Illumina DNA Prep Kit (Illumina Inc., San Diego, CA, USA), and samples were sequenced on the Illumina NovaSeq 6000 (Illumina Inc., San Diego, CA, USA). Paired-end reads (2 × 150) were trimmed using Trimmomatic-0.39 [18]. Trimmed reads were de novo assembled with MEGAHITv.1.2.9 [19]. Diamond [20] BLASTx was used to analyze the assembled contigs against a viral RefSeq database (release 207; downloaded from NCBI in September 2021). Circular genomes were determined based on the terminal redundancy in the de novo assembled contigs.

In the case of redondoviruses, the five genomes were recovered (for verification of de novo assemblies) via PCR amplification with Kapa HiFi HotStart ReadyMix DNA polymerase (Roche Diagnostics, Indianapolis, IN, USA) using abutting primer pairs designed based on the de novo assembled contigs. These amplicons were cloned into pJet1.2 plasmid vector (ThermoFisher Scientific, Waltham, MA, USA) and Sanger sequenced by primer walking at Macrogen Inc. (Seoul, South Korea). Redondovirus D_HF1_1 (OR148956) and D_HF5_1 (OR148962) were amplified using primer pair Re1_F/R (5′-GGGGCTACTTCTTTACAGGCAA-3′; 5′-ATCAACGGGTACTGTTCACTACCA-3′), redondovirus D_HF5_2R (OR148963) with Re2_F/R (5′-ATCAGAAACAGGTGTCACTGG-3′; 5′-GGTACTATACCAGTATAGGAAG-3′) and redondovirus D_HF1_3 (OR148957); and D_HF7_3 (OR148964) with Re3_F/R (5′-ATTTGTATGGCTATAATCCCATACTTACGCCG-3′; 5′-AAGGAGGAAGAGGATTATCAGATCCAAC-3′).

Viral genomes were annotated using VIBRANT [21] (for the large phages, microviruses, and inoviruses) and CenoteTaker2 [22] (for all other viruses). All annotations were manually checked. Pairwise identity calculations were computed with the Sequence Demarcation Tool (SDT) v1.2 [23]. For large phages and inoviruses, virus intergenomic similarities were computed with VIRIDIC [24].

### 2.3. Distribution of Virus Genomes across the Samples

To identify the distribution of the viral genomes across samples, we first clustered viruses into virus operational taxonomic units (vOTUs) with a 98% identity using SDT v1.2 [23]. For each unique vOTU, we mapped the reads from the Illumina sequencing to a representative genome of each vOTU using BBMap [25].

### 2.4. Phylogenetic Analyses

#### 2.4.1. Anelloviruses

Genome sequences of viruses in the genera *Alphatorquevirus* and *Betatorquevirus* and representatives in *Gammatorquevirus* (to serve as the outgroup) of the *Anelloviridae* family were downloaded from GenBank in May 2023. The ORF1 gene from the available GenBank sequences along with the ORF1 gene of anelloviruses identified in this study were extracted and translated. ORF1 amino acid sequences were aligned using MAFFT v.7.113 [26]. The alignment was used to infer a maximum likelihood phylogenetic tree using PhyML 3.0 [27] with best-fit amino acid substitution model VT+F determined using ProtTest 3 [28]. Branches with <0.7 approximate likelihood-ratio test (aLRT) branch support values were collapsed with TreeGraph2 [29].

#### 2.4.2. Cressdnaviruses

To determine the family-level assignment of the cressdnaviruses, the replication-associated protein (Rep) sequences were extracted from the nine genomes identified in this study and analyzed together with a dataset of Rep proteins of representative cressdnaviruses in the families *Bacillidnaviridae, Circoviridae, Geminiviridae, Genomoviridae, Metaxyviridae, Nanoviridae, Naryaviridae, Nenyaviridae, Redondoviridae, Smacoviridae*, and *Vilyaviridae*, as well as CRESS groups 1–6 [30] and those of *Alphasatellitidae*. The Rep sequences were used to generate a sequence similarity network (SSN) using EFI-EST [31] with a sequence similarity score of 60. Cytoscape V3.8.2 [32] was used to visualize the resulting SSN. A similarity threshold of 60 has previously demonstrated family-level groupings for cressdnaviruses [33,34,35,36,37,38,39,40].

We extracted the Rep sequences that form clusters with those from this study as well as those from the established viral cressdnavirus families and the CRESS groups 1–6. The sequences in this dataset were aligned with MAFFT v7.113 [26], and the alignment was trimmed with TrimAL (0.2 gap threshold) [41]. The trimmed alignment of the Rep amino acid sequences was then used to infer a maximum likelihood phylogenetic tree with IQ-TREE 2 [42] (with Q.pfam+F+G4 as the best-fit amino acid substitution model) and aLRT branch support [43]. The phylogenetic tree was visualized with iTOL v6 [44].

For each cluster from the SSN that had Rep sequences of the viruses identified in this study, we aligned the Rep amino acid sequences using MAFFT v7.113 [26]. This alignment was used to infer maximum likelihood phylogenetic trees using PhyML 3.0 [27] with best-fit models determined using ProtTest 3 [28] (LG+I+G+F for CRESS6, RtREV+I+G+F for Cluster 2, and LG+I+G+F for Cluster 1). Branches with <0.8 aLRT support were collapsed with TreeGraph2 [29].

#### 2.4.3. Microviruses

Complete genomes of microviruses available in GenBank were downloaded in May 2023. From the genomes and microvirus genomes identified in this study, major capsid protein (MCP) sequences were extracted. These, together with representative MCPs from members of the *Bullavirinae* sub-family (to serve as an outgroup), were translated and aligned with MAFFT v7.113 [26]. The alignment was then trimmed with TrimAL [41] (0.2 gap threshold) and used to infer a maximum likelihood phylogenetic tree using IQTree 2 [42] (with Q.pfam+F+G4 as the best-fit amino acid substitution model). The tree was visualized using iTOL v6 [44].

#### 2.4.4. Large Bacteriophages and Inoviruses

For the four large bacteriophages identified in this study, a proteomic tree of dsDNA bacteriophages was generated with ViPTree server version 3.1 (with auto gene prediction) [45]. For the inoviruses identified in this study, a custom database was generated of complete inovirus genomes available through GenBank, and this was used to infer a proteomic tree using ViPTree server version 3.1 (with auto gene prediction). Once the closest neighbors to the viruses identified in this study were determined, intergenomic distances within clades were calculated using VIRIDIC [24]. CheckV [46] was used to verify the completeness of annotated phage genomes.

## 3. Results and Discussion

**Figure 1:**
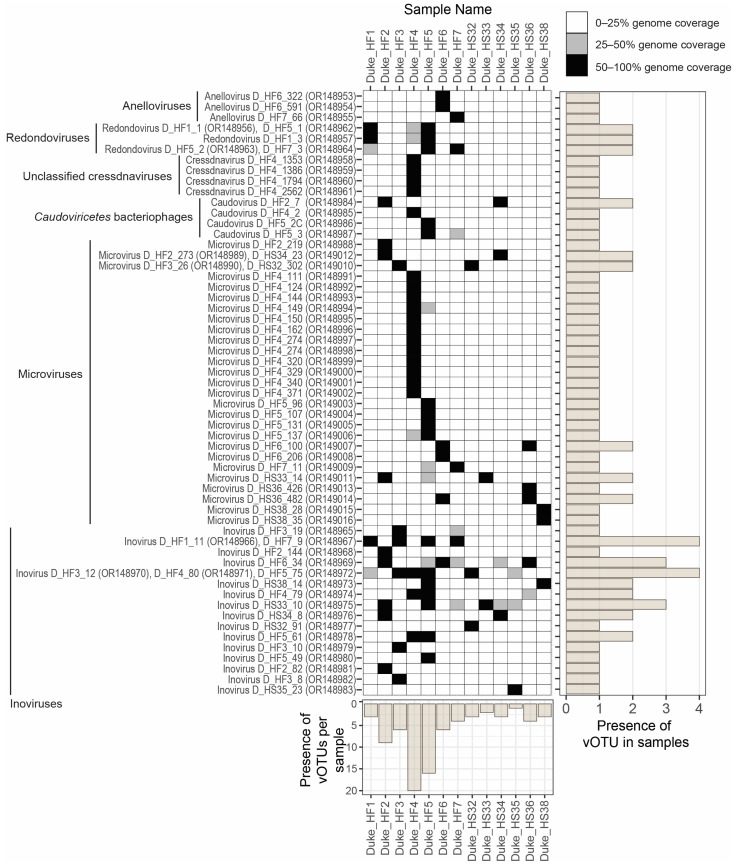
Genome coverage plot based on read mapping to vOTUs of viruses identified in this study depicting the presence of all vOTUs across all 14 human saliva samples. Black squares represent 50–100% genome coverage, gray squares represent 25–50% genome coverage, and white squares represent 0–25% genome coverage. Greater than 50% read coverage was used as a high-confidence proxy of vOTU presence in any particular sample. The bar plot on the right depicts the number of samples that each vOTU is present in. The bar plot at the bottom of the plot represents the number of vOTUs present in each saliva sample examined.

Our passive drool sampling approach of the saliva coupled with viral metagenomic workflows for DNA viruses resulted in the identification of genomes of diverse anelloviruses (*n* = 3), cressdnaviruses (*n* = 9), *Caudoviricetes* bacteriophages (*n* = 4), microviruses (*n* = 29), and inoviruses (*n* = 19) from human saliva. The viral genomes identified in this study and their accession numbers are summarized in Table 1. Human reads have been removed from all SRA-deposited data. The SRA-deposited data consist only of mapped reads to the viral described in this study. In 14 saliva samples from healthy individuals, we were able to obtain the complete genomes of viruses representing 55 species. The highest number of vOTUs were present (>50% genome coverage) in Duke_HF4 (*n* = 20), followed by Duke_HF5 (*n* = 16) and Duke_HF2 (*n* = 9) (Figure 1). The rest of the samples contained less than seven vOTUs each. Duke_HF4 contained vOTUs from unclassified cressdnaviruses (*n* = 4), *Caudoviricetes* bacteriophages (*n* = 1), microviruses (*n* = 12), and inoviruses (*n* = 3).

The vOTU consisting of inovirus D_HF1_11 (OR148966) and inovirus D_HF7_9 (OR148967) was present in four samples (Figure 1). The vOTU consisting of inovirus D_HF3_12 (OR148970), inovirus D_HF4_80 (OR148971), and inovirus D_HF5_75 (OR148972) was additionally present in four samples. Eight of the inovirus vOTUs were present in more than one sample. Five of the microvirus vOTUs were present in two samples. All three of the redondovirus vOTUs were present in two samples.

### 3.1. Anelloviruses

The *Anelloviridae* family consists of non-enveloped DNA viruses that have a high prevalence across global avian and mammal populations [47,48]. Anelloviruses have been identified across diverse hosts, including humans, nonhuman primates, livestock, birds, and even in some invertebrates (likely derived from a blood meal) [48,49,50]. They have been identified in various host sample types, including tissue, blood, fecal, nasal, and saliva samples [50,51,52]. Anelloviruses are consistently found as a part of various mammal and avian viromes with common coinfections of multiple anelloviruses in one host [47]. Within the context of human anelloviruses, Spandole et al. (2015) estimated that over 90% of humans in some regions, including Russia, Japan, and Pakistan, carry anelloviruses [53]. While anelloviruses have been detected in immunocompromised patients and are an emerging biomarker of immune response and, specifically, organ transplant rejection, they have not been directly associated with pathological effects on their hosts [8].

Anelloviruses have circular, negative-sense ssDNA genomes ranging between 1.6–3.9 kb in length [49]. Mammal-infecting anellovirus genomes consist of 1 large ORF1 and 2–3 small ORFs along with a conserved noncoding GC-rich region [49,54]. ORF1, comprising ~60% of the anellovirus genome, encodes a capsid protein [55]. Anelloviruses demonstrate high conservation of ORF1, with ORF1 nucleotide sequence similarity used to determine new anellovirus species (69% species demarcation threshold) [49]. While the *Anelloviridae* family consists of 30 genera, viruses in the three most well-characterized genera are the primate-infecting *Alphatorquevirus*, *Betatorquevirus*, and *Gammatorquevirus* [49,55].

**Figure 2:**
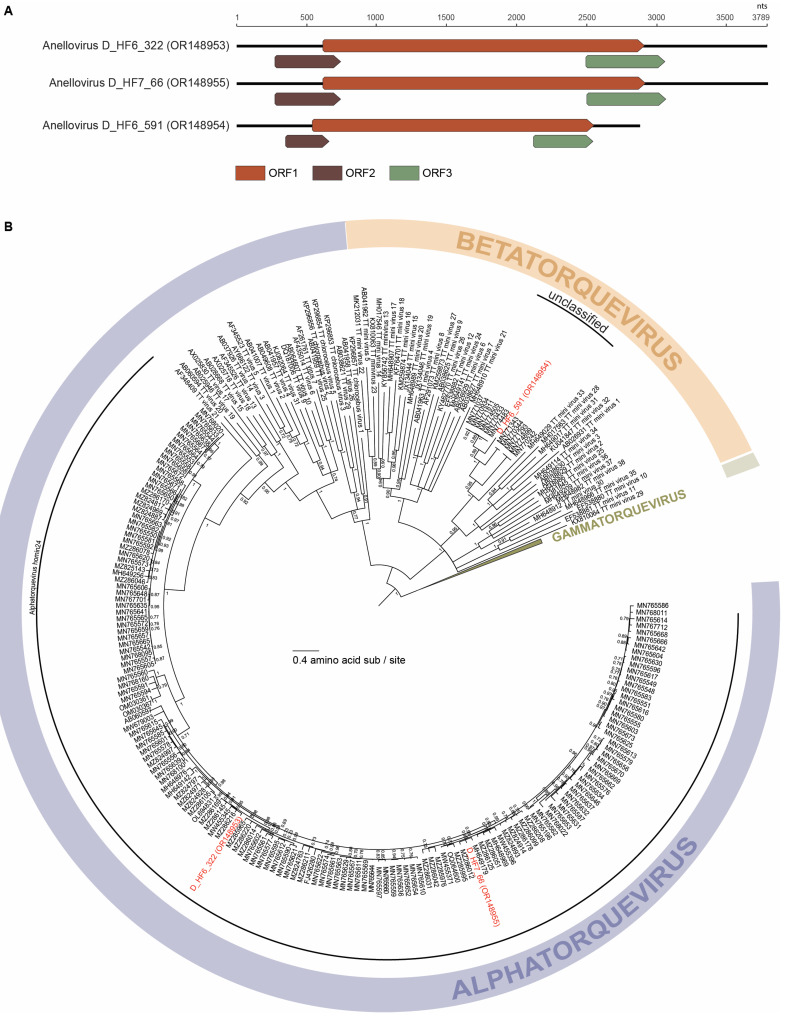
(**A**) Linearized genome organization of the three anellovirus genomes identified in this study. (**B**) Maximum likelihood phylogenetic tree of the ORF1 amino acid sequences of *Alphatorquevirus* and *Betatorquevirus* with *Gammatorquevirus* serving as the outgroup. aLRT branch support values are denoted by numbers at each node, and branches with values <0.7 aLRT branch support have been collapsed. Virus sequences identified in this study are in red font within the *Alphatorquevirus* and *Betatorquevirus* genera.

Three complete anellovirus genomes (Figure 2) were identified in this study. All three anelloviruses demonstrate the characteristic anellovirus genome architecture of a large ORF1 and two smaller ORFs, ORF2 and ORF3 (Figure 2). The anellovirus genomes identified in this study are 3794 nt (OR148953), 3017 nt (OR148954), and 3866 nt (OR148955) in length. These three anelloviruses have GC content ranging from 38.1% to 52.8%. Two of the anelloviruses, anellovirus D_HF6_322 (OR148953) and anellovirus D_HF6_591 (OR148954), were found to co-infect the same individual and share ~38% ORF1 nucleotide sequence similarity. The third anellovirus, anellovirus D_HF7_66 (OR148955), shares ~91% ORF1 nucleotide sequence similarity with anellovirus D_HF6_322 (OR148953). Phylogenetically, the three anelloviruses fall into two genera: *Alphatorquevirus* (anellovirus D_HF6_322, OR148953 and anellovirus D_HF7_66, OR148955) and *Betatorquevirus* (anellovirus D_HF6_591, OR148954) (Figure 2). Based on phylogenetic and pairwise identity analyses, anellovirus D_HF6_322 (OR148953) fits within a smaller lineage of four alphatorqueviruses with which it shares ~96% of ORF1 identity. Anellovirus D_HF6_322 (OR148953) shares ~70–94% of ORF1 nucleotide identity with the rest of *Alphatorquevirus*. Anellovirus D_HF7_66 (OR148955) is most closely related to a lineage of 17 alphatorqueviruses with which it shares ~96% of ORF1 nucleotide identity. Anellovirus D_HF7_66 (OR148955) shares ~71–93% of ORF1 nucleotide identity with alphatorqueviruses outside of its lineage. Anellovirus D_HF6_322 (OR148953) and anellovirus D_HF7_66 (OR148955) are members of the species *Alphatorquevirus homin24* (Figure 2). Anellovirus D_HF6_591 (OR148954) falls within a lineage of six betatorqueviruses with which it shares ~76% ORF1 sequence similarity (Figure 2). Anellovirus D_HF6_591 (OR148954) shares <67% ORF1 sequence similarity with betatorqueviruses outside of this lineage; thus, this new lineage represents a new species. All other viruses in this lineage have been identified from blood samples of children with febrile illness in Tanzania [56].

The complete genomes presented in this study add to the known diversity of anelloviruses and highlight that new lineages of anelloviruses are still being identified through viral metagenomics approaches. They additionally contribute to our understanding of anelloviruses’ global prevalence, specifically, across the eastern USA.

### 3.2. Cressdnaviruses

*Cressdnaviricota* is a rapidly growing yet elusive phylum consisting of diverse and globally distributed viruses [57]. The viruses within the phylum *Cressdnaviricota* infect eukaryotic hosts, including animals, fungi, plants, protists, and potentially archaea [6,58]. *Cressdnaviricota* currently contains 12 families of circular, replication-associated protein encoding single-stranded (CRESS) DNA viruses (*Amesuviridae*, *Bacilladnaviridae*, *Circoviridae, Geminiviridae, Genomoviridae, Metaxyviridae, Nanoviridae, Naryaviridae, Nenyaviridae, Redondoviridae, Smacoviridae, and Vilyaviridae*) [58,59]. In addition to viruses classified into these 12 genera, a large number of cressdnaviruses have been discovered that are yet to be placed within a defined family. The viruses in the phylum *Cressdnaviricota* are united by their small ssDNA genomes encoding a replication-associated protein (Rep) and capsid protein (Cp). As the Rep is more conserved across cressdnaviruses, this is generally utilized for phylogenetic analyses coupled with pairwise sequence identities for family- and genus-level classifications.

**Figure 3:**
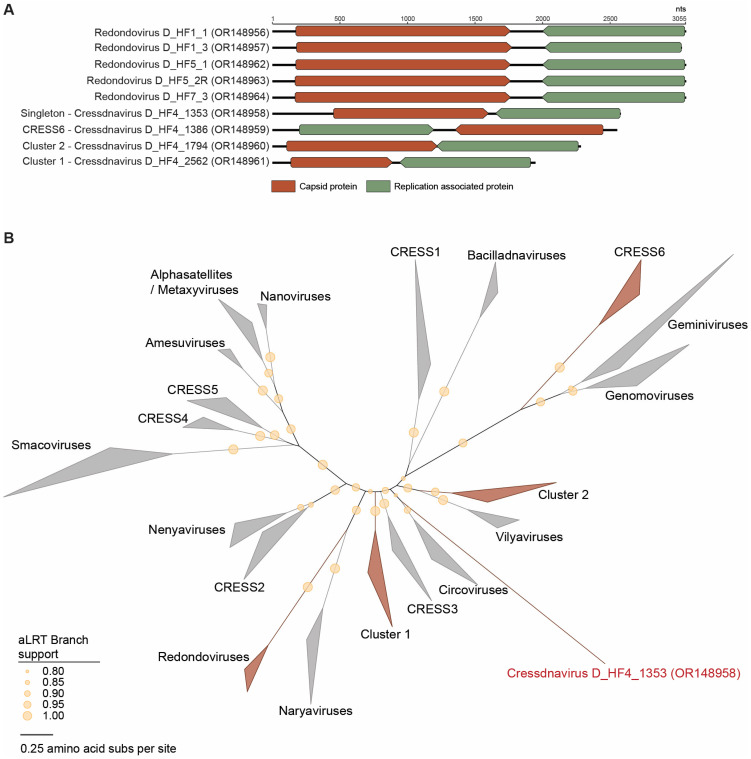
(**A**) Linearized genome organization of the cressdnaviruses identified in this study. (**B**) Maximum likelihood phylogenetic tree of the Rep sequences of viruses in the phylum *Cressdnaviricota* separated into family-level clustering. Family-level clusters that include viruses characterized in this study are colored in red. Cressdnavirus D_HF4_1353 (OR148958), an unclassified cressdnavirus depicted as its own line, falls within *Cressdnaviricota* but does not fit within the current family-level clusters. Branches with aLRT branch support values <0.8 were collapsed.

Nine genomes (Figure 3) that encode Rep were identified in this study. These genomes all fall within the *Cressdnaviricota* phylum based on their Rep analysis (Figure 3). Of these nine, five are part of the family *Redondoviridae*, three form clusters with various unclassified cressdnaviruses, and one is a singleton based on the Rep-based sequence similarity network and corresponding phylogeny (Figure 3). All cressdnavirus genomes identified in this study encode a Cp and Rep in a bidirectional orientation (Figure 3).

#### 3.2.1. Redondoviruses

Members of the family *Redondoviridae* were first identified in the human oro-respiratory tract. *Redondoviridae* is one of the more recently established and highly divergent families within *Cressdnaviricota* [60,61]. Redondoviruses are circular, ssDNA viruses with ~3–3.1 kb genomes [58]. The *Redondoviridae* family consists of one genus, *Torbevirus*, and two species, *Brisavirus* and *Vientovirus*, and these viruses have been found in high prevalence within human populations with frequent infections of two or more redondoviruses in a single individual [62]. Kinsella et al. (2022), using a computational workflow and over a thousand metagenomic datasets, predicted redondoviruses’ host to be *Entamoeba gingivalis*, an oral protozoan with an enigmatic role in periodontitis [6]. This was later confirmed by DNA proximity-ligation assay (Hi-C) on xenic culture cells [63].

**Figure 4:**
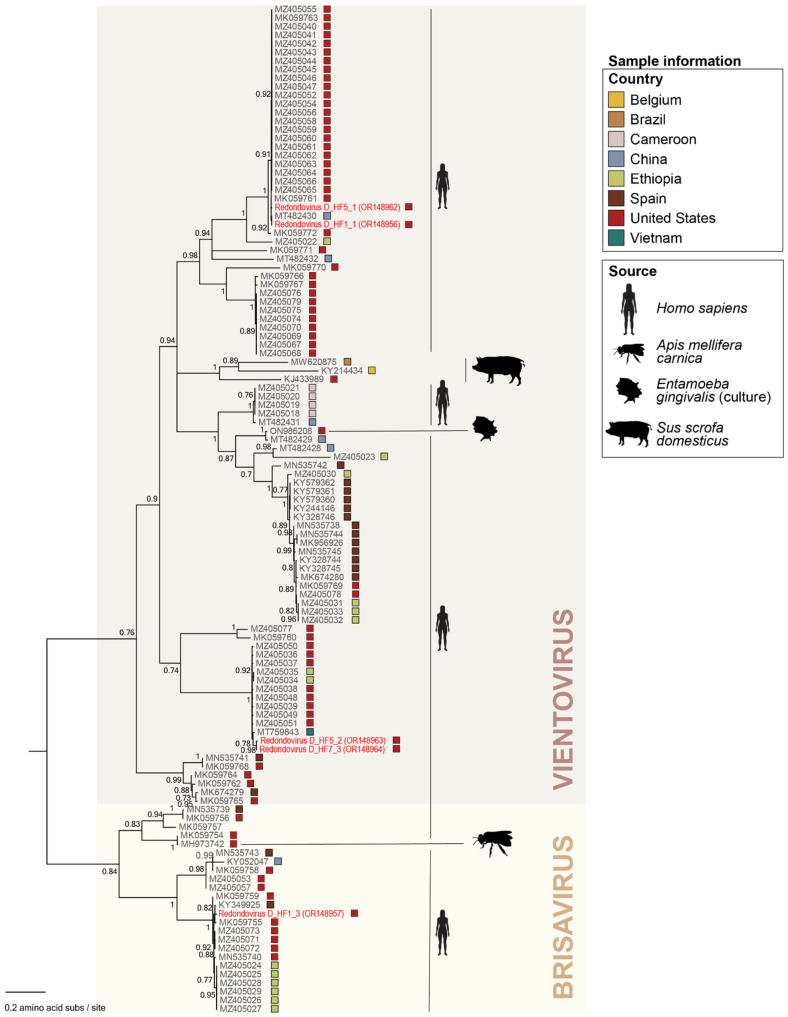
Maximum likelihood phylogenetic tree of the Rep amino acid sequences of viruses in the family *Redondoviridae*. The two species in the genus *Torbevirus* of the family *Redondoviridae*, *Brisavirus* and *Vientovirus*, are in shaded rectangles. Sample information including country in which the sample was taken and source the sample was collected from is depicted in the figure with colored squares next to each accession number and source silhouettes. aLRT branch support values are denoted by numbers at each node, and branches with values <0.7 were collapsed. Virus sequences identified in this study are highlighted in red font. Please refer to Figure 3B to view the placement of redondoviruses in the Rep-based cressdnavirus phylogenetic tree.

The five members of the *Redondoviridae* family identified here are 3055 nt (OR148956, OR148962, OR148963, OR148964) and 3024 nt (OR148957) in length (Figure 3). All have a GC content of 33.2% to 34% with redondovirus D_HF1_1 (OR148956) and redondovirus D_HF1_3 (OR148957) present in the same individual (Duke_HF1) and redondovirus D_HF5_1 (OR148962) and redondovirus D_HF5_2R (OR148963) present in another individual (Duke_HF5). Four of the identified redondoviruses, redondovirus D_HF1_1 (OR148956), redondovirus D_HF5_1 (OR148962), redondovirus D_HF5_2R (OR148963), and redondovirus D_HF7_3 (OR148964), fall within the *Vientovirus* species, while one redondovirus, D_HF1_3 (OR148957), falls within the *Brisavirus* species (Figure 4). Redondovirus D_HF5_2R (OR148963) and redondovirus D_HF7_3 (OR148964) share 99.6% of Rep nucleotide identity, and they fall within a lineage of 12 vientoviruses found across the USA, Vietnam, and Ethiopia with which they share ~99% of Rep nucleotide identity (Figure 4). The Reps of redondovirus D_HF1_1 (OR148956) and redondovirus D_HF5_1 (OR148962) phylogenetically form a clade of 25 vientoviruses with which they share >99% of Rep nucleotide identity (Figure 4). These vientoviruses have been detected across the USA and China. Redondovirus D_HF1_3 (OR148957) shares >98% of Rep nucleotide identity with its brisavirus lineage of 14 viruses. Redondoviruses within this brisavirus lineage have been characterized in the USA, Ethiopia, and Spain (Figure 4).

Despite the close genetic similarity between the redondoviruses characterized in this study and known redondoviruses, this work still adds to a growing body of knowledge about the prevalence of redondoviruses and highlights the success of using viral metagenomics and passive drool techniques to recover complete redondovirus genomes.

#### 3.2.2. Unclassified Cressdnaviruses

The phylum *Cressdnaviricota* is a recently established phylum and has 12 established families. Nonetheless, there are many cressdnaviruses that still remain unclassified and that represent new families and species [58]. Using sequence similarity networks of Rep proteins of cressdnaviruses, unclassified cressdnaviruses may belong to putative family-level clusters, such as CRESS1-6 and Clusters 1 and 2 (Figure 3). In this study, we identified four novel unclassified cressdnaviruses in one individual’s saliva sample (D_HF4). The genomes were 2546 nt (OR148959), 1938 nt (OR148961), 2279 nt (OR148960), and 2572 nt (OR148958) in length. These four unclassified cressdnaviruses have GC contents of 39.2% to 56.6%.

**Figure 5:**
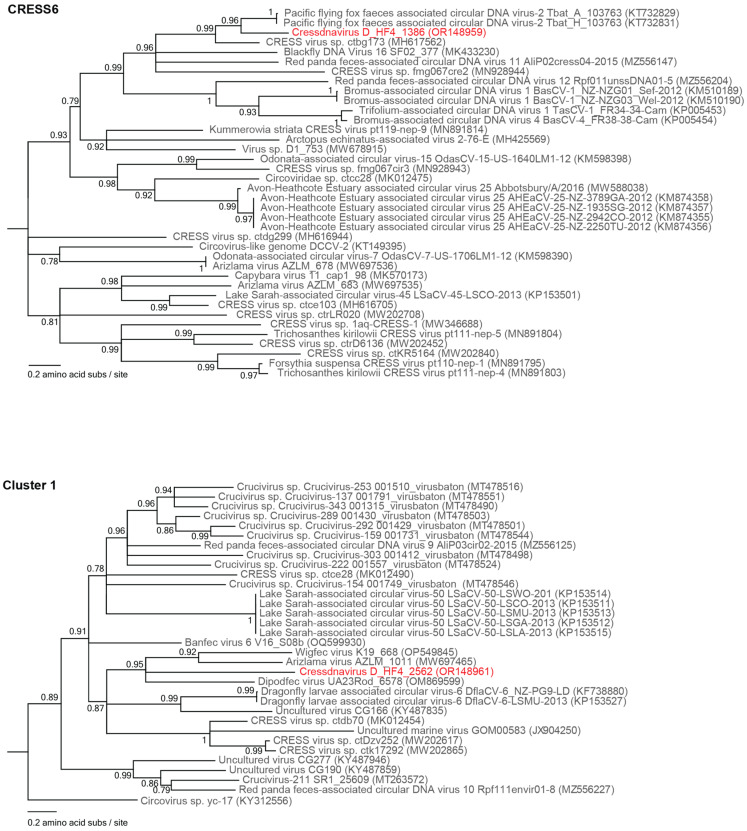
Maximum likelihood phylogenetic tree of the Rep sequences of unclassified family-level clusters within the phylum *Cressdnaviricota*, i.e., CRESS6 and Cluster 1 (Figure 3). Virus sequences identified in this study are highlighted in red font. Branches with <0.7 aLRT support have been collapsed. Please refer to Figure 3B to view the placement of CRESS6 and Cluster 1 in the Rep-based cressdnavirus phylogenetic tree.

The Reps of cressdnavirus D_HF4_1386 (OR148959) and cressdnavirus D_HF4_2562 (OR148961) fall within CRESS6 and Cluster 1, respectively (Figure 3 and Figure 5). The Rep of cressdnavirus D_HF4_1386 (OR148959) shares 66% of amino acid identity with that of Pacific flying fox feces-associated circular DNA virus-2 Tbat_A_103763 (KT732829) and Tbat_H_103763 (KT732831), both identified from flying fox fecal samples from the Kingdom of Tonga [64]. The Rep of cressdnavirus D_HF4_1386 (OR148959) shares ~30–60% of amino acid identity with those in all other lineages within CRESS6. The Rep of cressdnavirus D_HF4_2562 (OR148961) shares ~43–48% of amino acid identity with that of Dipodfec virus UA23Rod_6578 (OM869599) identified from a Banner-tailed kangaroo rat fecal sample [40], Arizlama virus AZLM_1011 (MW697465) from a lake water sample, and Wigfec virus K19_668 (OP549845) from an American wigeon sample, all sampled in Arizona (USA). In comparison with the Reps of other members of Cluster 1, it shares ~36–42% of amino acid identity.

**Figure 6:**
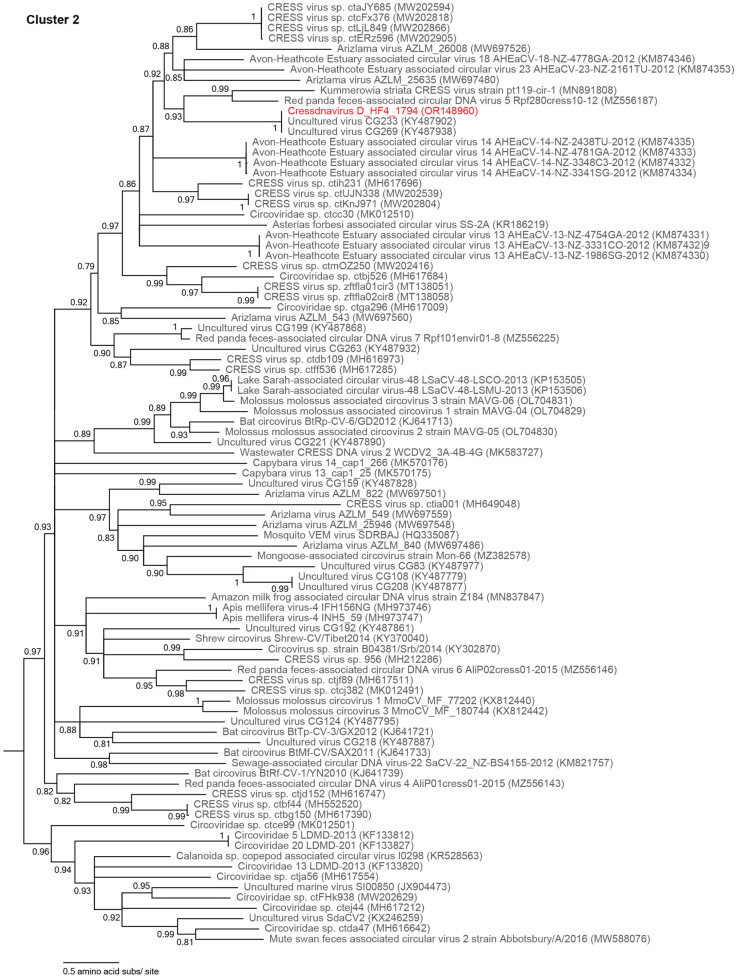
Maximum likelihood phylogenetic tree of the Rep sequences of an unclassified cluster within *Cressdnaviricota*, Cluster 2 (Figure 3). The virus characterized in this study is depicted in red font. Branches with <0.7 aLRT support have been collapsed. Please refer to Figure 3B to view the placement of Cluster 2 in the Rep-based cressdnavirus phylogenetic tree.

The Rep of one unclassified cressdnavirus, cressdnavirus D_HF4_1794 (OR148960), is part of Cluster 2 (Figure 6). The Rep of cressdnavirus D_HF4_1794 (OR148960) falls within a lineage with Reps of two other viruses, uncultured virus CG233 (KY487902) and CG269 (KY487938), with which it shares >99% of amino acid identity. Uncultured viruses CG233 (KY487902) and CG269 (KY487938) are both from wastewater samples from Florida, USA [65].

Additionally, the Rep of one cressdnavirus, cressdnavirus D_HF4_1353 (OR148958), cannot be placed within any of the current family-level clusters (Figure 3). Based on an NCBI BLASTp search of the Rep of cressdnavirus D_HF4_1353 (OR148958), it is most closely related to the Rep of *Cressdnaviricota* sp. Miresoil virus 60 (OM154761), sharing 37% of amino acid identity (query cover 77%). Cressdnaviricota sp. Miresoil virus 60 (OM154761) is a cressdnavirus identified from bog soil in Sweden [66].

The complete cressdnavirus genomes identified in this study highlight the significant number of novel cressdnaviruses present in just a limited number of samples from healthy individuals. Even within the saliva sample of one individual (D_HF4), cressdnaviruses are impressively diverse. Further, as in the case of cressdnavirus D_HF4_1794 (OR148960) sharing almost 100% amino acid identity with viruses recovered from wastewater, indirect detection of human-associated cressdnaviruses in wastewater is possible.

### 3.3. Tailed dsDNA Phages

The dsDNA bacteriophages in the viral class *Caudoviricetes* are the most abundant, diverse group of viruses on the earth, identified from Antarctic soils to the human gut, vaginal, and oral viromes [67,68,69,70]. Aside from the conserved major capsid protein with the characteristic HK97-fold, genomes of the members of *Caudoviricetes* vary in structure and size drastically [71]. For example, large bacteriophages can have dsDNA genomes ranging from ~18 kb encoding ~20–30 genes (*Roundtreeviridae, Salasmaviridae* viral families) to ~626 kb encoding hundreds of genes (*Bacillus* phage G; unassigned family) [72]. While there have been extensive studies on bacteriophages in the human gut virome, we are only beginning to unravel the diversity of bacteriophages in the oral virome [67]. Although this work presents a small number of genomes, these novel genomes add to bacteriophage diversity and will aid in the building of taxonomic frameworks for classification and association with oral bacteria [73,74].

**Figure 7:**
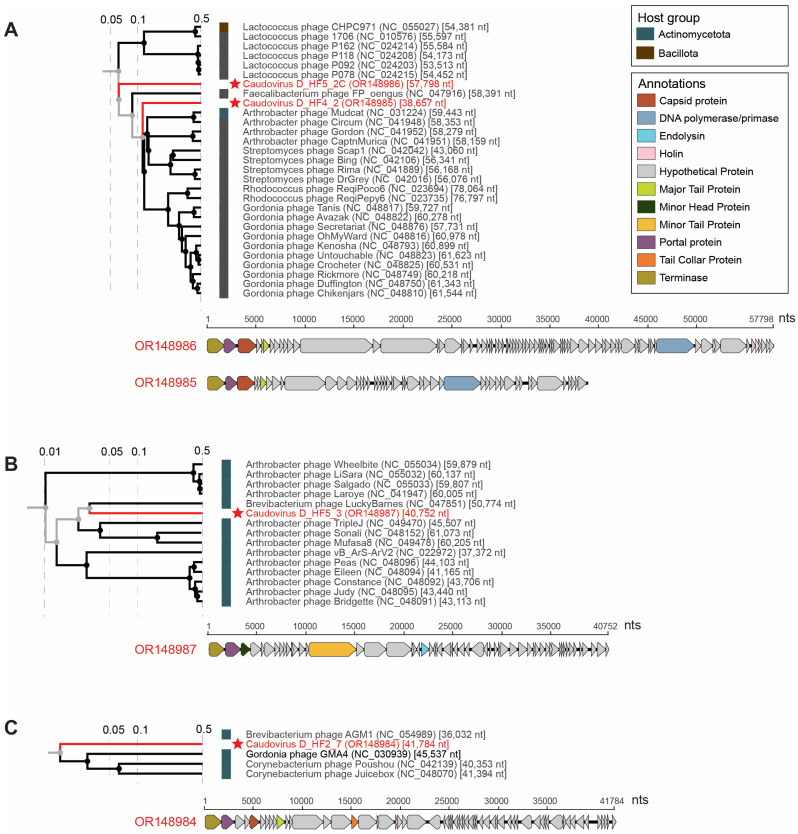
Proteomic trees and annotations of dsDNA phage genomes. The large phages characterized in this study fall primarily in three clades labeled (**A**–**C**). Virus genomes identified in this study are highlighted in red font and starred. The bacterial host group that the phage is predicted to infect is specified in color to the left of the accession information of each included virus. Genome annotations are depicted below each proteomic tree.

Four complete genomes of viruses in the class *Caudoviricetes* were identified in this study, and each represents a novel species. The predicted hosts of three of the large phages, caudovirus D_HF2_7 (OR148984), caudovirus D_HF4_2 (OR148985), and caudovirus D_HF5_3 (OR148987), are bacteria within the Actinomycetota phylum (Figure 7). Actinomycetota groups have been found to be important members of both the human microbiome and soil ecosystems [75,76]. Actinomyces are commensal, filamentous bacteria present across the various niches of the human microbiome. Actinomyces are able to induce actinomycosis, a rare granulomatous chronic disease primarily impacting immunocompromised people [76]. The fourth novel large phage caudovirus D_HF5_2C (OR148986) is predicted to infect bacteria in the Bacillota phylum (Figure 7). Bacillota, a phylum comprised of over 200 bacterial genera, has been found to consistently be one of the dominant groups in many human gut microbiome studies [77,78].

The genomes of these bacteriophages are 38,657 nt (OR148985), 57,798 nt (OR148986), 40,752 nt (OR148987), and 41,784 nt (OR148984) in length and encode numerous proteins, including capsid, terminase, portal, tail, and head proteins (Figure 7). These four genomes have a GC content of 37.3% to 61.1%. Caudovirus D_HF5_2C (OR148986) and caudovirus D_HF5_3 (OR148987) were present in the same individual (D_HF5). The four large phages fall primarily into three clades depicted as A, B, and C based on the ViPTree analysis (Figure 7). Caudovirus D_HF4_2 (OR148985) and caudovirus D_HF5_2C (OR148986) fall into Clade A (Figure 7). Caudovirus D_HF5_3 falls into Clade B, while caudovirus D_HF2_7 falls into Clade C (Figure 7). Analyzed through VIRIDIC, caudovirus D_HF4_2 (OR148985) shares <12%, caudovirus D_HF5_2C (OR148986) shares <7%, caudovirus D_HF5_3 (OR148987) shares <4%, and caudovirus D_HF2_7 (OR148984) shares <2% intergenomic similarity with the phages of Clades A, B and C, respectively (Figures S1–S3). As seen in the low intergenomic similarities between the *Caudoviricetes* bacteriophages identified in this study compared to known *Caudoviricetes* bacteriophages, caudoviruses are extraordinarily diverse with much of the large bacteriophage diversity remaining uncharacterized even within the human body.

### 3.4. Microviruses

Microviruses are small ssDNA bacteriophages known to infect enterobacteria and to be relatively ubiquitous across metagenomic surveys [33,79,80,81]. Microvirus genomes are ~4–6 kb and usually contain multiple overlapping reading frames. Their genomes typically encode a more conserved major capsid protein (MCP) along with a replication initiator protein (Rep) and scaffolding proteins [33,82]. The *Microviridae* family is currently composed of two sub-families, *Gokushovirinae* and *Bullavirinae* [80]. However, recent research emphasizing the diversity of microviruses has suggested that the current *Microviridae* family should be elevated to its own order comprising 3 suborders and 19 families [80]. These extensive, potential taxonomic adjustments reveal that our knowledge base of microviruses has rapidly expanded and will continue to do so with the rise of viral metagenomic methods.

**Figure 8:**
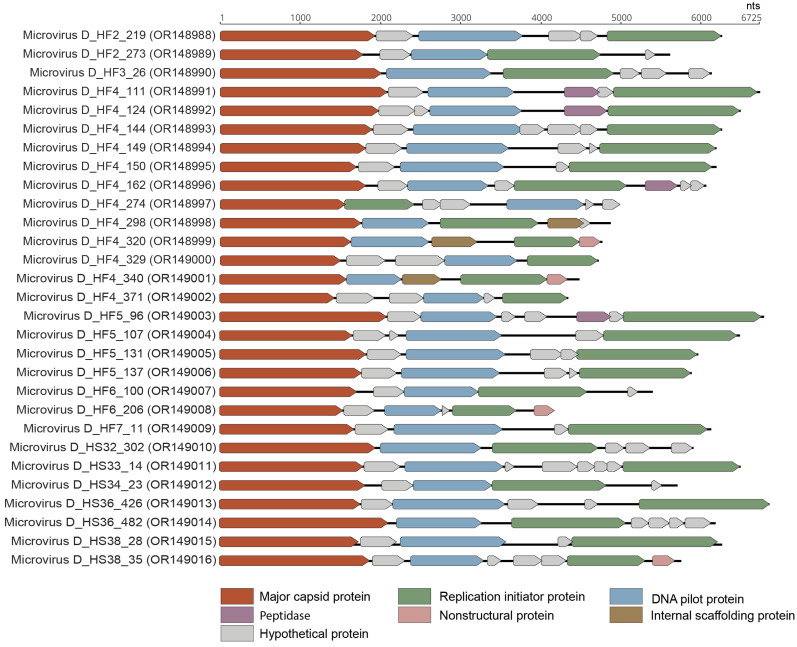
Linearized genome annotations of microviruses identified in this study.

**Figure 9:**
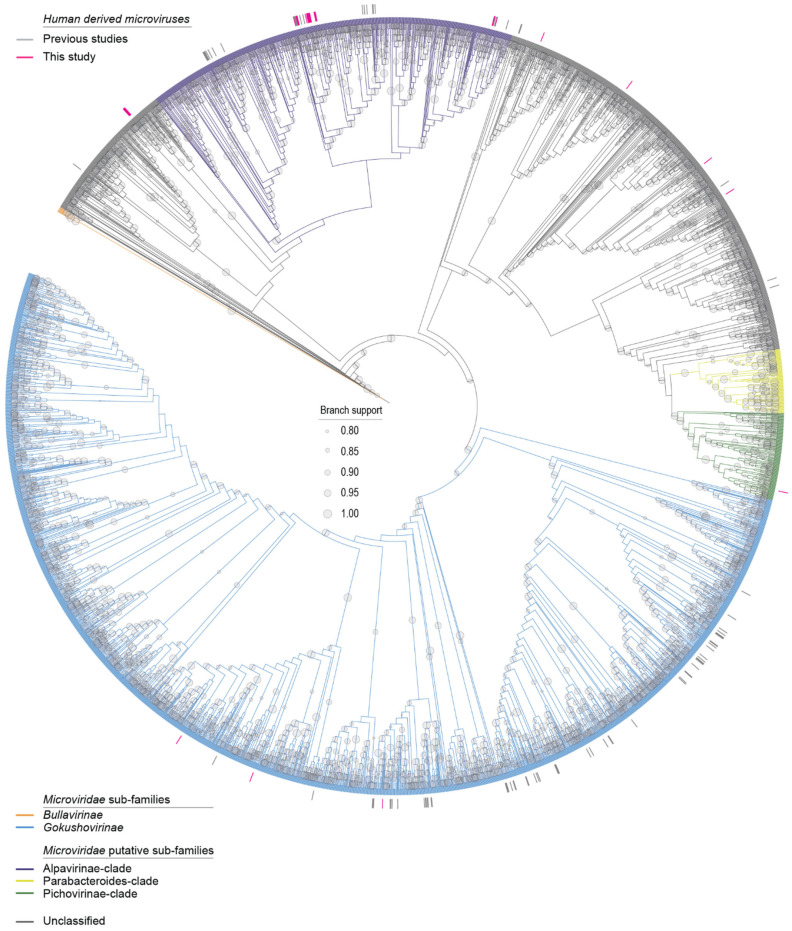
Maximum likelihood phylogenetic tree of the *Microviridae* family. The tree branches are colored by sub-families and putative sub-families. Human-derived microviruses from previous studies (gray) and this study (pink) are denoted as short lines around the outer edge of the circular phylogeny.

In total, 29 complete microvirus genomes were identified in this study representing 27 vOTUs. Twelve microvirus vOTUs were identified in one saliva sample (Duke_HF4) (Figure 1). The genomes ranged from 4311 to 7033 nt in length, and all the 29 microvirus genomes encode at least an MCP, Rep, and DNA pilot protein (Figure 8). These microviruses have a GC content of 32.7% to 56.6%. The microviruses identified in this study, based on the MCP, are phylogenetically located within the *Gokushovirinae* sub-family, Alpavirinae putative sub-family, and Pichivirinae putative sub-family (Figure 9) based on the MCP amino acid phylogeny. BLASTn analyses were performed to determine the similarity of the microviruses identified in this study to previously characterized microviruses. The 29 microviruses share 70% to 99% of nucleotide identity to known microvirus sequences with a query cover ranging from 3% to 100% (Table 2). Many of these microviruses share high similarity with sequences identified from human metagenome studies [10] (*n* = 21). Two of the identified microviruses, microvirus D_HF4_150 (OR148995) and microvirus D_HS33_14 (OR149011), share >95% nucleotide sequence similarity with microviruses identified by Tisza et al. (2021), denoting that they are the same species as previously characterized microviruses [10]. Microvirus D_HF4_150 (OR148995) and microvirus D_HS33_14 (OR149011) share 99% (query cover 100%) and 97% of nucleotide identity (query cover 93%) with Microviridae sp. cti0q21 (BK051052) and Microviridae sp.ctMkX8 (BK042793), respectively, both from a human oral sample with predicted bacterial genus host *Prevotella* [10]. *Prevotella*, an anaerobic Gram-negative bacterium, has been found to be abundant in the human oral cavity, particularly in the subgingival plaque [83], and although most strains have low pathogenicity, some have been associated with chronic inflammatory diseases [84]. The other microviruses share 70–84% of nucleotide identity with sequences of microviruses from the human nasopharyngeal cavity (*n* = 1), tortoise feces (*n* = 1), minnow tissue (*n* = 3), freshwater (*n* = 1), wastewater (*n* = 1), and a tunicate intestinal tract (*n* = 1).

Overall, the microviruses described here infect bacteria, such as *Prevotella*, residing in the human oral cavity. Although the importance of microviruses in the human gut has been previously emphasized [80], the diversity of microviruses shown here from only 14 saliva samples demonstrates the prominence of microviruses in the human oral cavity. As microviruses infect bacteria that both play a commensal role in the human microbiome and have pathogenic potential, microviruses are important members of the human oral virome, likely controlling the abundance and behavior of their bacterial hosts.

### 3.5. Inoviruses

The *Inoviridae* family consists of diverse, filamentous bacteriophages known to infect hosts across the Bacteria domain and potentially across Archaea [85]. Viruses in the family *Inoviridae* are classified into 25 genera with 43 species [86]. Inoviruses have a 5.5–10.6 kb circular ssDNA genome [86]. The inovirus genome replicates via a rolling-circle mechanism and encodes 7–15 proteins [86]. Inoviruses have the capability to integrate themselves into host genomes and cause chronic infection cycles [85]. Additionally, inoviruses can directly and indirectly impact the toxicity of known pathogenic bacteria, including *Vibrio cholerae, Pseudomonas, Neisseria,* and *Ralstonia* [87]. A few specific inoviruses have been extensively studied and used in a variety of genetic engineering applications due to their smaller genome size and uniquely filamentous virion [85]. Yet, the majority of inoviruses remain uncharacterized as emphasized in the works of Roux et al. (2019) and Tisza et al. (2021) who discovered thousands of inovirus-like sequences across existing genomes and metagenomes [10,85]. Our work here adds to growing efforts to understand inovirus prevalence, diversity, and function in humans using metagenomics.

**Figure 10:**
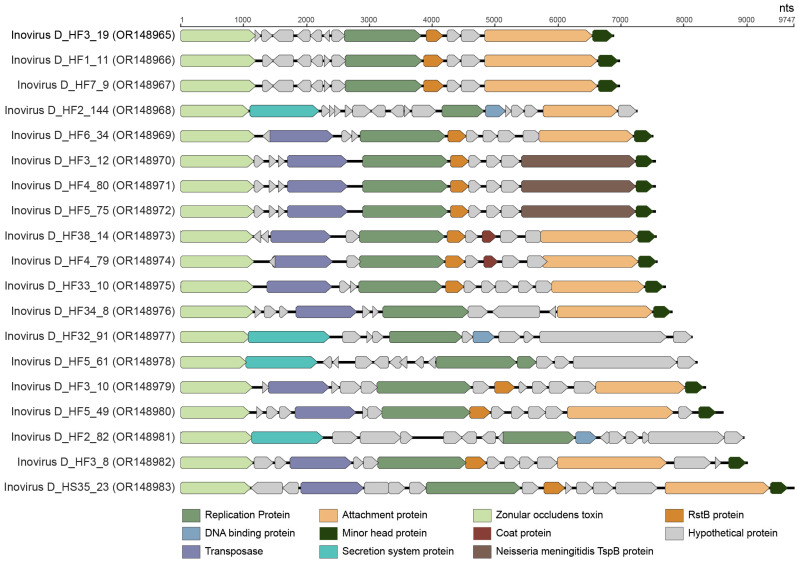
Linearized genome annotations of inoviruses identified in this study.

**Figure 11:**
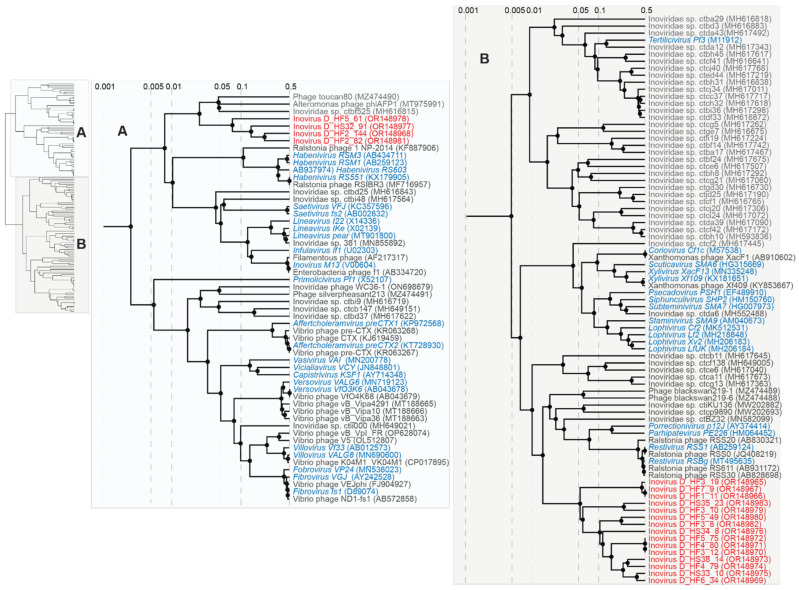
Proteomic tree of viruses in the *Inoviridae* family. Viruses have been placed into two distinct clades within the *Inoviridae* family, labeled as Clades (**A**) (blue) and (**B**) (green). Inoviruses identified in this study are highlighted in red font, and those that have been classified are highlighted in blue font with species names in italics.

Nineteen complete inovirus genomes were identified in this study with genomes ranging from 6884 to 9747 nt in length (Figure 10). All 19 inovirus genomes encode a replication protein and zonular occludens toxin, a morphogenesis protein essential for phage assembly [88]. The inoviruses identified in this study have GC contents of 33.9% to 48.5%. These inoviruses primarily fall into two clades of the *Inoviridae* family, depicted as Clades A and B in Figure 11. The 19 inoviruses form two novel, distinct lineages within their respective clades (Figure 11). Inovirus D_HF5_61 (OR148978), inovirus D_HS32_91 (OR148977), inovirus D_HF2_144 (OR148968), and inovirus D_HF2_82 (OR148981) form a lineage within Clade A. Inovirus D_HF2_82 (OR148981) and inovirus D_HF2_144 (OR148968) share ~33% intergenomic similarity as computed from VIRIDIC (Figure S6). Inovirus D_HF5_61 (OR148978) and inovirus D_HS32_91 (OR148977) share greater similarity with one another than the other inoviruses of Clade A; however, they still have <20% intergenomic similarity (Figure S4). The rest of the identified inoviruses (*n* = 15) form a distinct lineage within Clade B (Figure 11). Inovirus D_HF3_12 (OR148970), inovirus D_HF4_80 (OR148971), and inovirus D_HF5_75 (OR148972) share 100% intergenomic similarity, denoting that they are the same inovirus species (Figure S5). Additionally, inovirus D_HF1_11 (OR148966) and inovirus D_HF7_9 (OR148967) share >99% intergenomic similarity, denoting that they are the same inovirus species (Figure S5).

Based on BLASTn, all 19 inoviruses have the highest nucleotide sequence similarity, ranging from 72–100% nucleotide identity (with 21–100% query cover), with the inoviruses identified from the metagenomes of human oral samples [10]. Additionally, the predicted bacterial hosts, as determined in the work of Tisza et al. (2021), of the closest BLASTn hits of the 19 inoviruses identified in this study include the genera *Neisseria* (*n* = 13), *Aggregatibacter* (*n* = 3; Inovirus D_HF2_82, OR148981; inovirus D_HF2_144, OR148968; inovirus D_HS32_91, OR148977), and *Mannheimia* (*n* = 1; Inovirus D_HF5_61, OR148978) [10]. While most members of *Neisseria* are commensal, two species of the *Neisseria* genus are opportunistic pathogens responsible for cases of meningitis, septicemia, and gonorrhea in humans [89]. Bacteria within the *Neisseria* genus have iron-regulated proteins related to the RTX toxin superfamily [89,90]. Bacteria in the *Aggregatibacter* genus can contribute to periodontal disease, particularly in children and adolescents [91]. One of the main virulence factors of bacteria within *Aggregatibacter* is a leukotoxin capable of causing extensive damage to human immune tissues [91]. Although bacteria in the *Mannheimia* genus are infrequently associated with disease, some species can cause pneumonia and septicemia in domestic animals and have been isolated from septicemia and wound infections in humans [92]. Similar to *Aggregatibacter*, *Mannheimia*’s most important virulence factor is a leukotoxin able to cause cell lysis and death [93]. As previous studies have shown inoviruses’ ability to impact the toxicity of toxin-producing bacterial genera, inoviruses likely serve a crucial role in controlling the function of pathogenic and commensal bacteria of the human oral virome.

## 4. Conclusions

High-throughput sequencing and viral metagenomic workflows are innovative tools to help identify diverse novel and known viruses across complex viral phyla. In this study, we successfully de novo assembled 64 complete genomes of viruses across *Anelloviridae, Cressdnaviricota, Caudoviricetes, Microviridae,* and *Inoviridae*, representing 55 species in only 14 saliva samples from healthy individuals in Durham, North Carolina (USA). Two of the complete anellovirus genomes represent the species *Alphatorquevirus homin24*, and the third anellovirus falls within a lineage of unclassified betatorqueviruses. Four of the five redondoviruses are parts of the species *Vientovirus*, and one is part of *Brisavirus.* In sample Duke_HF1, we identified redondoviruses, redondovirus D_HF1_1 (OR148956) and redondovirus D_HF1_3 (OR148957), which are members of two species, and in sample Duke_HF5, we identified two variants, redondovirus D_HF5_1 (OR148962) and redondovirus D_HF5_2R (OR148963), of the species *Vientovirus*. This shows that multiple variants and species of redodonviruses are circulating in individuals.

All four unclassified cressdnaviruses represent new species within *Cressdnaviricota*. One unclassified cressdnavirus, cressdnavirus D_HF4_1794 (OR148960), has high similarity with uncultured virus CG233 (KY487902) and CG269 (KY487938) identified from a wastewater sample from Florida (USA) [65], showing that this virus is shed via the fecal route. All four new unclassified cressdnaviruses were identified in a single saliva sample (Duke_HF4). Four *Caudoviricetes* phages were identified, all representing new species. The 29 microvirus genomes show high BLASTn similarity to microvirus sequences from human metagenomes, the human nasopharyngeal cavity, tortoise feces, minnow tissue, freshwater, wastewater, and a tunicate intestinal tract. The nine genomes fall into lineages of the ViPTree-generated proteome phylogenetic tree.

Overall, the number of complete virus genomes determined, with many of these viruses representing new species, shows that the human oral virome is relatively understudied. Although many of these viruses infect microbes within the human body and not human cells, viruses such as *Caudoviricetes* phages, microviruses, and inoviruses (which can impact the toxicity of bacterial pathogens) are likely key influencers of bacterial abundance and behavior within the human oral cavity. These bacteriophages are directly impacting bacteria that influence human immunity and health. The discovery of known and new viruses in microbe-infecting phyla is therefore vital for understanding how widespread that impact may be. For all the viral phyla and families studied, we are only beginning to understand their vast diversity and global prevalence even within the human body. The work presented here significantly contributes to our global understanding of viruses in the human oral virome and displays the surprising levels of novel viral diversity that can be revealed from 14 saliva samples. Lastly, this study supports the utility of passive drool sampling in conjunction with metagenomics for effective discovery across diverse viral groups.

## Figures and Tables

**Table 1:** Overview of viruses characterized by this study. All viral genomes were deposited in GenBank with accession numbers as shown below.

Phylum	Class	Family	Number of Virus Genomes Identified	GenBank Accession Number
N/A	N/A	*Anelloviridae*	3	OR148953, OR148954, OR148955
*Cressdnaviricota*	*Arfiviricetes*	*Redondoviridae*	5	OR148956, OR148957, OR148962, OR148963, OR148964
	N/A	Unclassified	4	OR148958, OR148959, OR148960, OR148961
*Uroviricota*	*Caudoviricetes*	N/A	4	OR148984, OR148985, OR148986, OR148987
*Phixviricota*	*Malgrandaviricetes*	*Microviridae*	29	OR148988, OR148989, OR148990, OR148991, OR148992, OR148993, OR148994, OR148995, OR148996, OR148997, OR148998, OR148999, OR149000, OR149001, OR149002, OR149003, OR149004, OR149005, OR149006, OR149007, OR149008, OR149009, OR149010, OR149011, OR149012, OR149013, OR149014, OR149015, OR149016
*Hofneiviricota*	*Faserviricetes*	*Inoviridae*	19	OR148965, OR148966, OR148967, OR148968, OR148969, OR148970, OR148971, OR148972, OR148973, OR148974, OR148975, OR148976, OR148977, OR148978, OR148979, OR148980, OR148981, OR148982, OR148983

**Table 2:** Summary of microviruses identified in this study and their percent identity to the closest related microvirus according to BLASTn.

Accession Number	Genome Length (nt)	Data for Top BLASTn Hit			
		Best Hit (NCBI Accession No.)	Percent Identity (%)	Query Cover (%)	Microvirus Source
**OR148988**	6252	BK022849	80	89	Human metagenome, USA
**OR148989**	5600	BK050880	93	91	Human metagenome, USA
**OR148990**	6125	BK047429	74	6	Human metagenome, USA
**OR148991**	6725	BK053955	83	62	Human metagenome, USA
**OR148992**	6482	BK040340	92	100	Human metagenome, USA
**OR148993**	6250	BK022849	78	74	Human metagenome, USA
**OR148994**	6185	BK044794	93	91	Human metagenome, USA
**OR148995**	6178	BK051052	99	100	Human metagenome, USA
**OR148996**	6058	BK031150	73	33	Human metagenome, USA
**OR148997**	4966	MH572463	72	21	*Ciona robusta* intestinal tract, USA
**OR148998**	4861	MT310328	71	39	Wastewater metagenome, USA
**OR148999**	4753	MH617740	72	96	Minnow tissue metagenome. USA
**OR149000**	4708	MH617589	71	19	Minnow tissue metagenome, USA
**OR149001**	4645	MT204053	70	18	Freshwater metagenome, Svalbard
**OR149002**	4499	MH617140	76	38	Minnow tissue metagenome, USA
**OR149003**	7033	BK041046	72	53	Human metagenome, USA
**OR149004**	6719	BK015704	94	100	Human metagenome, USA
**OR149005**	6177	BK022849	78	80	Human metagenome, USA
**OR149006**	6102	BK044794	89	91	Human metagenome, USA
**OR149007**	5590	BK050880	92	91	Human metagenome, USA
**OR149008**	4311	BK039346	75	84	Human metagenome, USA
**OR149009**	6346	BK039742	91	100	Human metagenome, USA
**OR149010**	6125	BK047429	74	6	Human metagenome, USA
**OR149011**	6378	BK042793	97	93	Human metagenome, USA
**OR149012**	5601	BK050880	93	91	Human metagenome, USA
**OR149013**	6729	BK049539	73	95	Human metagenome, USA
**OR149014**	6067	MG883710	84	92	Human nasopharyngeal cavity, China
**OR149015**	6152	BK053938	90	90	Human metagenome, USA
**OR149016**	5654	MK765647	70	3	Tortoise feces, USA

## Data Availability

The sequences described in this study were deposited in GenBank under accession numbers OR148953–OR149016. The raw reads were deposited in SRA under BioProject number: PRJNA956591; BioSample numbers: SAMN35328316–SAMN35328328; SRA accession: SRR24738781–SRR24738793.

## Supplementary Materials

The following supporting information can be downloaded at: https://www.mdpi.com/article/10.3390/v15091821/s1, Figure S1: Viridic heatmap of *Caudoviricetes* bacteriophage Clade A; Figure S2: Viridic heatmap of *Caudoviricetes* bacteriophage Clade B; Figure S3: Viridic heatmap of *Caudoviricetes* bacteriophage Clade C; Figure S4: Viridic heatmap of inovirus Clade A.; Figure S5: Viridic heatmap of *Caudoviricetes* bacteriophages.

## References

[1] Liang G., Bushman F.D. (2021). The human virome: Assembly, composition and host interactions. Nat. Rev. Genet..

[2] Shaiber A., Willis A.D., Delmont T.O., Roux S., Chen L.-X., Schmid A.C., Yousef M., Watson A.R., Lolans K., Esen Ö.C. (2020). Functional and genetic markers of niche partitioning among enigmatic members of the human oral microbiome. Genome Biol..

[3] Li S., Guo R., Zhang Y., Li P., Chen F., Wang X., Li J., Jie Z., Lv Q., Jin H. (2022). A catalog of 48,425 nonredundant viruses from oral metagenomes expands the horizon of the human oral virome. iScience.

[4] Ho S.X., Min N., Wong E.P.Y., Chong C.Y., Chu J.J.H. (2021). Characterization of oral virome and microbiome revealed distinctive microbiome disruptions in paediatric patients with hand, foot and mouth disease. npj Biofilms Microbiomes.

[5] Abeles S.R., Ly M., Santiago-Rodriguez T.M., Pride D.T. (2015). Effects of Long Term Antibiotic Therapy on Human Oral and Fecal Viromes. PLoS ONE.

[6] Kinsella C.M., Deijs M., Becker C., Broekhuizen P., van Gool T., Bart A., Schaefer A.S., van der Hoek L. (2022). Host prediction for disease-associated gastrointestinal cressdnaviruses. Virus Evol..

[7] Guidry J.T., Birdwell C.E., Scott R.S. (2018). Epstein-Barr virus in the pathogenesis of oral cancers. Oral Dis..

[8] Arze C.A., Springer S., Dudas G., Patel S., Bhattacharyya A., Swaminathan H., Brugnara C., Delagrave S., Ong T., Kahvejian A. (2021). Global genome analysis reveals a vast and dynamic anellovirus landscape within the human virome. Cell Host Microbe.

[9] Abeles S.R., Robles-Sikisaka R., Ly M., Lum A.G., Salzman J., Boehm T.K., Pride D.T. (2014). Human oral viruses are personal, persistent and gender-consistent. ISME J..

[10] Tisza M.J., Buck C.B. (2021). A catalog of tens of thousands of viruses from human metagenomes reveals hidden associations with chronic diseases. Proc. Natl. Acad. Sci. USA.

[11] Fernandes A., Skinner M.L., Woelfel T., Carpenter T., Haggerty K.P. (2012). Implementing Self-Collection of Biological Specimens with a Diverse Sample. Field Methods.

[12] Ng D.P., Koh D., Choo S.G., Ng V., Fu Q. (2004). Effect of storage conditions on the extraction of PCR-quality genomic DNA from saliva. Clin. Chim. Acta.

[13] Oskis A., Loveday C., Hucklebridge F., Thorn L., Clow A. (2009). Diurnal patterns of salivary cortisol across the adolescent period in healthy females. Psychoneuroendocrinology.

[14] Robles-Sikisaka R., Ly M., Boehm T., Naidu M., Salzman J., Pride D.T. (2013). Association between living environment and human oral viral ecology. ISME J..

[15] Willner D., Furlan M., Schmieder R., Grasis J.A., Pride D.T., Relman D.A., Angly F.E., McDole T., Mariella R.P., Rohwer F. (2011). Metagenomic detection of phage-encoded platelet-binding factors in the human oral cavity. Proc. Natl. Acad. Sci. USA.

[16] DiGiulio D.B., Callahan B.J., McMurdie P.J., Costello E.K., Lyell D.J., Robaczewska A., Sun C.L., Goltsman D.S.A., Wong R.J., Shaw G. (2015). Temporal and spatial variation of the human microbiota during pregnancy. Proc. Natl. Acad. Sci. USA.

[17] Goltsman D.S.A., Sun C.L., Proctor D.M., DiGiulio D.B., Robaczewska A., Thomas B.C., Shaw G.M., Stevenson D.K., Holmes S.P., Banfield J.F. (2018). Metagenomic analysis with strain-level resolution reveals fine-scale variation in the human pregnancy microbiome. Genome Res..

[18] Bolger A.M., Lohse M., Usadel B. (2014). Trimmomatic: A flexible trimmer for Illumina sequence data. Bioinformatics.

[19] Li D., Liu C.-M., Luo R., Sadakane K., Lam T.-W. (2015). MEGAHIT: An ultra-fast single-node solution for large and complex metagenomics assembly via succinct de Bruijn graph. Bioinformatics.

[20] Buchfink B., Xie C., Huson D.H. (2015). Fast and sensitive protein alignment using DIAMOND. Nat. Methods.

[21] Kieft K., Zhou Z., Anantharaman K. (2020). VIBRANT: Automated recovery, annotation and curation of microbial viruses, and evaluation of viral community function from genomic sequences. Microbiome.

[22] Tisza M.J., Belford A.K., Domínguez-Huerta G., Bolduc B., Buck C.B. (2021). Cenote-Taker 2 democratizes virus discovery and sequence annotation. Virus Evol..

[23] Muhire B.M., Varsani A., Martin D.P. (2014). SDT: A Virus Classification Tool Based on Pairwise Sequence Alignment and Identity Calculation. PLoS ONE.

[24] Moraru C., Varsani A., Kropinski A.M. (2020). VIRIDIC—A Novel Tool to Calculate the Intergenomic Similarities of Prokaryote-Infecting Viruses. Viruses.

[25] Bushnell B. (2015). BBMap Short-Read Aligner, and Other Bioinformatics Tools. http://sourceforge.net/projects/bbmap/.

[26] Katoh K., Standley D.M. (2013). MAFFT Multiple Sequence Alignment Software Version 7: Improvements in Performance and Usability. Mol. Biol. Evol..

[27] Guindon S., Dufayard J.-F., Lefort V., Anisimova M., Hordijk W., Gascuel O. (2010). New Algorithms and Methods to Estimate Maximum-Likelihood Phylogenies: Assessing the Performance of PhyML 3.0. Syst. Biol..

[28] Darriba D., Taboada G.L., Doallo R., Posada D. (2011). ProtTest 3: Fast selection of best-fit models of protein evolution. Bioinformatics.

[29] Stöver B.C., Müller K.F. (2010). TreeGraph 2: Combining and visualizing evidence from different phylogenetic analyses. BMC Bioinform..

[30] Kazlauskas D., Varsani A., Krupovic M. (2018). Pervasive Chimerism in the Replication-Associated Proteins of Uncultured Single-Stranded DNA Viruses. Viruses.

[31] Zallot R., Oberg N., Gerlt J.A. (2019). The EFI Web Resource for Genomic Enzymology Tools: Leveraging Protein, Genome, and Metagenome Databases to Discover Novel Enzymes and Metabolic Pathways. Biochemistry.

[32] Shannon P., Markiel A., Ozier O., Baliga N.S., Wang J.T., Ramage D., Amin N., Schwikowski B., Ideker T. (2003). Cytoscape: A software environment for integrated models of Biomolecular Interaction Networks. Genome Res..

[33] Chrzastek K., Kraberger S., Schmidlin K., Fontenele R.S., Kulkarni A., Chappell L., Dufour-Zavala L., Kapczynski D.R., Varsani A. (2021). Diverse Single-Stranded DNA Viruses Identified in Chicken Buccal Swabs. Microorganisms.

[34] Custer J.M., White R., Taylor H., Schmidlin K., Fontenele R.S., Stainton D., Kraberger S., Briskie J.V., Varsani A. (2021). Diverse single-stranded DNA viruses identified in New Zealand (Aotearoa) South Island robin (*Petroica australis*) fecal samples. Virology.

[35] Fontenele R.S., Lacorte C., Lamas N.S., Schmidlin K., Varsani A., Ribeiro S.G. (2019). Single Stranded DNA Viruses Associated with Capybara Faeces Sampled in Brazil. Viruses.

[36] Harding C., Larsen B.B., Otto H.W., Potticary A.L., Kraberger S., Custer J.M., Suazo C., Upham N.S., Worobey M., Van Doorslaer K. (2023). Diverse DNA virus genomes identified in fecal samples of Mexican free-tailed bats (*Tadarida brasiliensis*) captured in Chiricahua Mountains of southeast Arizona (USA). Virology.

[37] Kraberger S., Schmidlin K., Fontenele R.S., Walters M., Varsani A. (2019). Unravelling the Single-Stranded DNA Virome of the New Zealand Blackfly. Viruses.

[38] Levy H., Fontenele R.S., Harding C., Suazo C., Kraberger S., Schmidlin K., Djurhuus A., Black C.E., Hart T., Smith A.L. (2020). Identification and Distribution of Novel Cressdnaviruses and Circular Molecules in Four Penguin Species in South Georgia and the Antarctic Peninsula. Viruses.

[39] Lund M.C., Larsen B.B., Rowsey D.M., Otto H.W., Gryseels S., Kraberger S., Custer J.M., Steger L., Yule K.M., Harris R.E. (2023). Using archived and biocollection samples towards deciphering the DNA virus diversity associated with rodent species in the families cricetidae and heteromyidae. Virology.

[40] Orton J.P., Morales M., Fontenele R.S., Schmidlin K., Kraberger S., Leavitt D.J., Webster T.H., Wilson M.A., Kusumi K., Dolby G.A. (2020). Virus Discovery in Desert Tortoise Fecal Samples: Novel Circular Single-Stranded DNA Viruses. Viruses.

[41] Capella-Gutiérrez S., Silla-Martínez J.M., Gabaldón T. (2009). trimAl: A tool for automated alignment trimming in large-scale phylogenetic analyses. Bioinformatics.

[42] Minh B.Q., Schmidt H.A., Chernomor O., Schrempf D., Woodhams M.D., von Haeseler A., Lanfear R. (2020). IQ-TREE 2: New Models and Efficient Methods for Phylogenetic Inference in the Genomic Era. Mol. Biol. Evol..

[43] Anisimova M., Gascuel O. (2006). Approximate Likelihood-Ratio Test for Branches: A Fast, Accurate, and Powerful Alternative. Syst. Biol..

[44] Letunic I., Bork P. (2021). Interactive Tree of Life (iTOL) v5: An online tool for phylogenetic tree display and annotation. Nucleic Acids Res..

[45] Nishimura Y., Yoshida T., Kuronishi M., Uehara H., Ogata H., Goto S. (2017). ViPTree: The viral proteomic tree server. Bioinformatics.

[46] Nayfach S., Camargo A.P., Schulz F., Eloe-Fadrosh E., Roux S., Kyrpides N.C. (2020). CheckV assesses the quality and completeness of metagenome-assembled viral genomes. Nat. Biotechnol..

[47] Kaczorowska J., Deijs M., Klein M., Bakker M., Jebbink M.F., Sparreboom M., Kinsella C.M., Timmerman A.L., van der Hoek L. (2022). Diversity and Long-Term Dynamics of Human Blood Anelloviruses. J. Virol..

[48] Kraberger S., Opriessnig T., Celer V., Maggi F., Okamoto H., Blomström A.-L., Cadar D., Harrach B., Biagini P., Varsani A. (2021). Taxonomic updates for the genus *Gyrovirus* (family Anelloviridae): Recognition of several new members and establishment of species demarcation criteria. Arch. Virol..

[49] Varsani A., Opriessnig T., Celer V., Maggi F., Okamoto H., Blomström A.-L., Cadar D., Harrach B., Biagini P., Kraberger S. (2021). Taxonomic update for mammalian anelloviruses (family Anelloviridae). Arch. Virol..

[50] Okamoto H. (2009). TT Viruses in Animals.

[51] Bigarré L., Beven V., de Boisséson C., Grasland B., Rose N., Biagini P., Jestin A. (2005). Pig anelloviruses are highly prevalent in swine herds in France. J. Gen. Virol..

[52] Collins C.L., Kraberger S., Fontenele R.S., Faleye T.O.C., Adams D., Adhikari S., Sandrolini H., Finnerty S., Halden R.U., Scotch M. (2022). Genome Sequences of Anelloviruses, Genomovirus, and Papillomavirus Isolated from Nasal Pharyngeal Swabs. Genome Announc..

[53] Spandole S., Cimponeriu D., Berca L.M., Mihăescu G. (2015). Human anelloviruses: An update of molecular, epidemiological and clinical aspects. Arch. Virol..

[54] Amatya R., Deem S.L., Porton I.J., Wang D., Lim E.S. (2017). Complete Genome Sequence of Torque teno indri virus 1, a Novel Anellovirus in Blood from a Free-Living Lemur. Genome Announc..

[55] Butkovic A., Kraberger S., Smeele Z., Martin D.P., Schmidlin K., Fontenele R.S., Shero M.R., Beltran R.S., Kirkham A.L., Aleamotu’a M. (2023). Evolution of anelloviruses from a circovirus-like ancestor through gradual augmentation of the jelly-roll capsid protein. Virus Evol..

[56] Cordey S., Laubscher F., Hartley M.-A., Junier T., Keitel K., Docquier M., Guex N., Iseli C., Vieille G., Le Mercier P. (2021). Blood virosphere in febrile Tanzanian children. Emerg. Microbes Infect..

[57] Krupovic M., Varsani A. (2021). A 2021 taxonomy update for the family *Smacoviridae*. Arch. Virol..

[58] Krupovic M., Varsani A., Kazlauskas D., Breitbart M., Delwart E., Rosario K., Yutin N., Wolf Y.I., Harrach B., Zerbini F.M. (2020). *Cressdnaviricota*: A Virus Phylum Unifying Seven Families of Rep-Encoding Viruses with Single-Stranded, Circular DNA Genomes. J. Virol..

[59] Krupovic M., Varsani A. (2022). Naryaviridae, Nenyaviridae, and Vilyaviridae: Three new families of single-stranded DNA viruses in the phylum *Cressdnaviricota*. Arch. Virol..

[60] Noell K., Kolls J.K. (2019). Further Defining the Human Virome using NGS: Identification of Redondoviridae. Cell Host Microbe.

[61] Abbas A.A., Taylor L.J., Dothard M.I., Leiby J.S., Fitzgerald A.S., Khatib L.A., Collman R.G., Bushman F.D. (2019). Redondoviridae, a Family of Small, Circular DNA Viruses of the Human Oro-Respiratory Tract Associated with Periodontitis and Critical Illness. Cell Host Microbe.

[62] Taylor L.J., Dothard M.I., Rubel M.A., Allen A.A., Hwang Y., Roche A.M., Graham-Wooten J., Fitzgerald A.S., Khatib L.A., Ranciaro A. (2021). Redondovirus Diversity and Evolution on Global, Individual, and Molecular Scales. J. Virol..

[63] Keeler E.L., Merenstein C., Reddy S., Taylor L.J., Cobián-Güemes A.G., Zankharia U., Collman R.G., Bushman F.D. (2023). Widespread, human-associated redondoviruses infect the commensal protozoan Entamoeba gingivalis. Cell Host Microbe.

[64] Male M.F., Kraberger S., Stainton D., Kami V., Varsani A. (2016). Cycloviruses, gemycircularviruses and other novel replication-associated protein encoding circular viruses in Pacific flying fox (*Pteropus tonganus*) faeces. Infect. Genet. Evol..

[65] Pearson V.M., Caudle S.B., Rokyta D.R. (2016). Viral recombination blurs taxonomic lines: Examination of single-stranded DNA viruses in a wastewater treatment plant. PeerJ.

[66] Trubl G., Roux S., Borton M.A., Varsani A., Li Y.F., Sun C., Jang H.B., Woodcroft B., Tyson G., Wrighton K. (2023). Population ecology and potential biogeochemical impacts of ssDNA and dsDNA soil viruses along a permafrost thaw gradient. bioRxiv.

[67] Pride D.T., Salzman J., Haynes M., Rohwer F., Davis-Long C., White R.A., Loomer P., Armitage G.C., Relman D.A. (2011). Evidence of a robust resident bacteriophage population revealed through analysis of the human salivary virome. ISME J..

[68] Zablocki O., van Zyl L., Adriaenssens E.M., Rubagotti E., Tuffin M., Cary S.C., Cowan D. (2014). High-Level Diversity of Tailed Phages, Eukaryote-Associated Viruses, and Virophage-Like Elements in the Metaviromes of Antarctic Soils. Appl. Environ. Microbiol..

[69] Adriaenssens E.M. (2021). Phage Diversity in the Human Gut Microbiome: A Taxonomist’s Perspective. mSystems.

[70] Happel A.-U., Varsani A., Balle C., Passmore J.-A., Jaspan H. (2020). The Vaginal Virome—Balancing Female Genital Tract Bacteriome, Mucosal Immunity, and Sexual and Reproductive Health Outcomes?. Viruses.

[71] Benler S., Yutin N., Antipov D., Rayko M., Shmakov S., Gussow A.B., Pevzner P., Koonin E.V. (2021). Thousands of previously unknown phages discovered in whole-community human gut metagenomes. Microbiome.

[72] González B., Monroe L., Li K., Yan R., Wright E., Walter T., Kihara D., Weintraub S.T., Thomas J.A., Serwer P. (2020). Phage G Structure at 6.1 Å Resolution, Condensed DNA, and Host Identity Revision to a Lysinibacillus. J. Mol. Biol..

[73] Turner D., Kropinski A.M., Adriaenssens E.M. (2021). A Roadmap for Genome-Based Phage Taxonomy. Viruses.

[74] Sommers P., Chatterjee A., Varsani A., Trubl G. (2021). Integrating Viral Metagenomics into an Ecological Framework. Annu. Rev. Virol..

[75] Benaud N., Chelliah D.S., Wong S.Y., Ferrari B.C. (2022). Soil substrate culturing approaches recover diverse members of Actinomycetota from desert soils of Herring Island, East Antarctica. Extremophiles.

[76] Li J., Li Y., Zhou Y., Wang C., Wu B., Wan J. (2018). Actinomyces and Alimentary Tract Diseases: A Review of Its Biological Functions and Pathology. BioMed Res. Int..

[77] Panwar D., Shubhashini A., Kapoor M. (2023). Complex alpha and beta mannan foraging by the human gut bacteria. Biotechnol. Adv..

[78] Lupu V.V., Raileanu A.A., Mihai C.M., Morariu I.D., Lupu A., Starcea I.M., Frasinariu O.E., Mocanu A., Dragan F., Fotea S. (2023). The Implication of the Gut Microbiome in Heart Failure. Cells.

[79] Collins C.L., DeNardo D.F., Blake M., Norton J., Schmidlin K., Fontenele R.S., Wilson M.A., Kraberger S., Varsani A. (2021). Genome Sequences of Microviruses Identified in Gila Monster Feces. Genome Announc..

[80] Kirchberger P.C., Martinez Z.A., Ochman H. (2022). Organizing the Global Diversity of Microviruses. mBio.

[81] Cherwa J.E., Fane B.A. (2011). Microviridae: Microviruses and Gokushoviruses. eLS.

[82] Uchiyama A., Chen M., Fane B.A. (2007). Characterization and Function of Putative Substrate Specificity Domain in Microvirus External Scaffolding Proteins. J. Virol..

[83] The Human Microbiome Project Consortium (2012). Structure, function and diversity of the healthy human microbiome. Nature.

[84] Larsen J.M. (2017). The immune response to Prevotella bacteria in chronic inflammatory disease. Immunology.

[85] Roux S., Krupovic M., Daly R.A., Borges A.L., Nayfach S., Schulz F., Sharrar A., Carnevali P.B.M., Cheng J.-F., Ivanova N.N. (2019). Cryptic inoviruses revealed as pervasive in bacteria and archaea across Earth’s biomes. Nat. Microbiol..

[86] Knezevic P., Adriaenssens E.M. (2021). ICTV Report Consortium ICTV Virus Taxonomy Profile: Inoviridae. J. Gen. Virol..

[87] Ilyina T.S. (2015). Filamentous bacteriophages and their role in the virulence and evolution of pathogenic bacteria. Mol. Genet. Microbiol. Virol..

[88] Loh B., Haase M., Mueller L., Kuhn A., Leptihn S. (2017). The Transmembrane Morphogenesis Protein gp1 of Filamentous Phages Contains Walker A and Walker B Motifs Essential for Phage Assembly. Viruses.

[89] Dworkin M., Falkow S., Rosenberg E., Schleifer K.-H., Stackebrandt E. (2006). The Prokaryotes: Volume 5: Proteobacteria: Alpha and Beta Subclasses.

[90] Thompson S.A., Wang L.L., West A., Sparling P.F. (1993). Neisseria meningitidis produces iron-regulated proteins related to the RTX family of exoproteins. J. Bacteriol..

[91] Gholizadeh P., Pormohammad A., Eslami H., Shokouhi B., Fakhrzadeh V., Kafil H.S. (2017). Oral pathogenesis of Aggregatibacter actinomycetemcomitans. Microb. Pathog..

[92] Corbel M.J., Greenwood D., Barer M., Slack R., Irving W. (2012). 35—Yersinia, Pasteurella and Francisella: Plague; Pseudotuberculosis; Mesenteric Adenitis; Pasteurellosis; Tularaemia. Medical Microbiology.

[93] Aulik N.A., Hellenbrand K.M., Klos H., Czuprynski C.J. (2010). *Mannheimia haemolytica* and its leukotoxin cause neutrophil extracellular trap formation by bovine neutrophils. Infect. Immun..

