# Supplementary material for: Characterization of Diverse Anelloviruses, Cressdnaviruses, and Bacteriophages in the Human Oral DNA Virome from North Carolina (USA)
Source: Viruses. 2023 Aug 26;15(9):1821. doi: 10.3390/v15091821 (PMC10537320; doi:10.3390/v15091821)
Supplement: Supplementary file 1 [file viruses-15-01821-s001.zip › Supplementary Figure 1_EP.pdf]

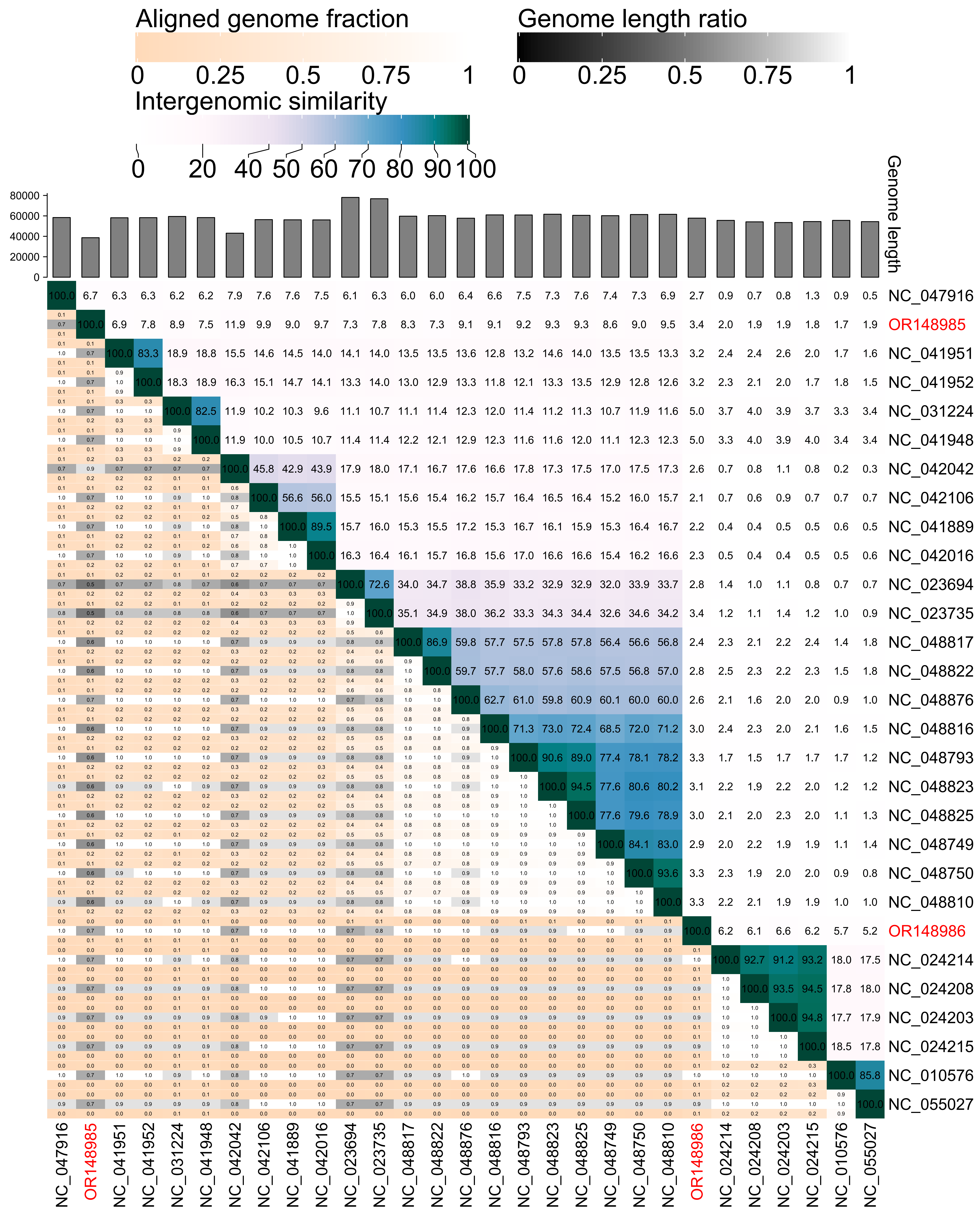


**Figure S1.** Heatmap of computed intergenomic distances amongst *Caudoviricetes* bacteriophage clade A (Figure 6) viral genomes using VIRIDIC.
