# Supplementary figures and images for: Characterization of Diverse Anelloviruses, Cressdnaviruses, and Bacteriophages in the Human Oral DNA Virome from North Carolina (USA)
Source: Viruses. 2023 Aug 26;15(9):1821. doi: 10.3390/v15091821 (PMC10537320; doi:10.3390/v15091821)

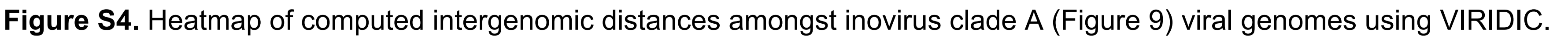

Supplement: Supplementary file 1 [file viruses-15-01821-s001.zip › Supplementary Figure 4_EP.pdf]

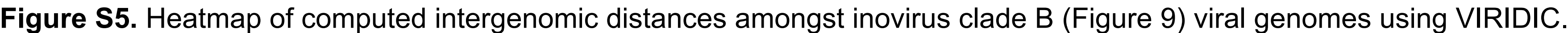

Supplement: Supplementary file 1 [file viruses-15-01821-s001.zip › Supplementary Figure 5_EP.pdf]
